# Preferable timing of intraductal ultrasonography during endoscopic retrograde cholangiopancreatography lithotomy: A prospective cohort study

**DOI:** 10.3389/fmed.2022.1042929

**Published:** 2022-10-26

**Authors:** Zhanjun Lu, Hang Zhao

**Affiliations:** ^1^Department of Gastroenterology, Shanghai General Hospital Jintan District Hospital, Jintan, China; ^2^Shanghai Municipal Hospital of Traditional Chinese Medicine, Shanghai, China

**Keywords:** intraductal ultrasonography, lithotomy, endoscopic retrograde cholangiopancreatography, common bile duct (CBD), stone

## Abstract

**Aim:**

Intraductal ultrasonography (IDUS) is a highly sensitive and non-invasive detective method that can be used to detect complete calculus clearance during endoscopic retrograde cholangiopancreatography (ERCP). In this study, we examined the preferable timing of IDUS during ERCP lithotomy.

**Methods:**

From 2017 to 2020, patients with choledocholithiasis were randomized into IDUS-BL (IDUS performed before lithotomy) group, IDUS-ALC (cholangiography and IDUS performed after lithotomy) group, and IDUS-AL group (IDUS performed after lithotomy) group. The influence of IDUS on the accuracy of prejudgment, the incidence of residual stones, the need for repeated lithotomy (RL), and fluoroscopy time were analyzed.

**Results:**

A total of 184 patients were enrolled. No residual stones were found during follow-up in any of the three groups. There was no difference in prejudgment accuracy rate on size and number of stones between different groups (all *P* > 0.05). RL were performed in 5, 9, and 9 cases of IDUS-BL, IDUS-ALC, and IDUS-AL group, respectively (*P* > 0.05). IDUS-AL group had a shorter fluoroscopy time than the other two groups (1.5 ± 0.6 vs. 2.8 ± 1.2, 2.5 ± 1.0 min, *P* < 0.05). Incidence of RL was related to the location of calculus [middle or lower part of common bile duct (CBD)], lithotripsy, dilated CBD (2.12 ± 0.46 vs. 1.78 ± 0.40 cm, *P* < 0.01), and inaccuracy prejudgment.

**Conclusion:**

IDUS performed after lithotomy is preferable for shorten fluoroscopy time during ERCP. IDUS is a reliable solution for the stone omission, which may be more valuable for patients with high-risk factors of RL.

## Introduction

Common bile duct (CBD) stone, also known as choledocholithiasis, is a common clinical disease associated with abnormal metabolism, old age, impaired biliary function, and abnormal anatomical structure (1). CBD is usually treated with endoscopic retrograde cholangiopancreatography (ERCP); the success rate of ERCP intubation can reach up to 98%, while the stone clearance rate after ERCP has been reported to range from 85 to 92% (2). Previous studies have suggested that ERCP is relatively safe, cost-effective, and associated with a low complication rate compared to other traditional open surgery or laparoscopic transcystic CBD exploration (2, 3). Various researchers have proposed proper indications of endoscopic lithotomy. For example, CBD patients with stones larger than 12 mm have the lowest rate of success and the highest rate of complications (4). Stricture in the lower part of CBD is another reason for unsuccessful clearance, which hinders the passing of stones with bigger diameters. Big stones usually need to be removed by choledochoscope, percutaneous puncture, or surgery (5). Moreover, recurrence of stones after ERCP removal also commonly occurs among certain patients. For example, the highest early recurrence rate within 1 year has been reported to be around 24% (2, 5, 6). Residual stones are an important reason for early recurrence, which occurs in a short period after ERCP. Accordingly, this may result in prolonged hospital stay in certain cases. According to statistics, the residual rate of stones can reach 12.9% (7, 8). Several factors contribute to the residual of stones, including the presence of multiple stones, debris from lithotripsy, and other stone-related characteristics (9). The inappropriate concentration of contrast and improper position of the patient may result in unclear cholangiography, which is also one of the reasons to perform the procedure (9).

Intraductal ultrasonography (IDUS) can provide real-time, high-quality cross-sectional images. IDUS has a higher diagnostic sensitivity for micro stones compared to computed tomography (CT), magnetic resonance imaging (MRI), and MRCP (10), as well as a high-resolution ability. The operator’s skills also influence the output of IDUS, especially when doing examination on the wall of the bile duct or the pancreas (11). Yet, so far, the value and effectiveness of IDUS on CBD calculus have been rarely reported. In this study, we performed a preliminary analysis of intervention timing and practical value of IDUS on lithotomy during ERCP.

## Materials and methods

### Patients

A total of 210 patients diagnosed with choledocholithiasis at Shanghai General Hospital between 2017 and 2020 were recruited. Inclusion criteria were: patients confirmed with single or multiple CBD stones. Exclusion criteria included intolerance of ERCP, post partial or total gastrectomy, inappropriate endoscopic lithotomy with a higher failure probability during evaluation, and accidental diagnosis of the tumor during ERCP. Finally, 184 cases were enrolled in our study. All patients were confirmed with single or multiple CBD stones by imaging method including abdominal ultrasonography (US), CT, and MRI. Written informed consent was obtained from each patient. The study protocol was approved by the Ethics Committees of the Shanghai General Hospital.

**FIGURE 1:**
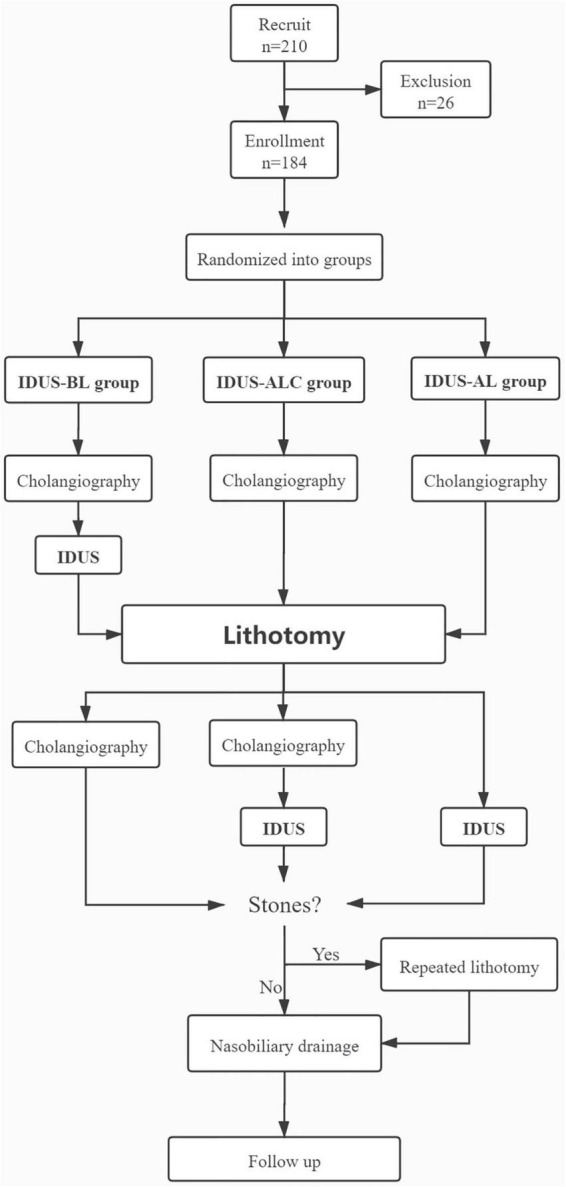
Consort flow diagram of patients undergoing ERCP and IDUS.

All patients were prospectively randomized into three groups by generated random numbers: IDUS-BL group (IDUS performed before lithotomy), IDUS-ALC group (cholangiography and IDUS performed after lithotomy), and IDUS-AL group (IDUS performed after lithotomy). Patients in different groups underwent different stone extraction procedural. After successful cannulation, cholangiography was performed in all three groups of patients. Then, the IDUS-BL group underwent IDUS before lithotomy. Next, lithotomies were performed in all groups. After extraction of stones, patients in the IDUS-BL group underwent cholangiography; the IDUS-ALC group underwent cholangiography and IDUS, while the IDUS-AL group only underwent IDUS. If the operator considered incomplete clearance of CBD either by cholangiography or by IDUS, repeated lithotomy (RL) was carried out. The summary flow process is shown in Figure 1. Total clearance rate and fluoroscopy time were carefully investigated according to the following methods.

### Equipment and anesthesia methods

Endoscopic retrograde cholangiopancreatography was performed using a standard side-viewing duodenoscope (TJF-260, Olympus, Tokyo, Japan). A 2.0-mm-diameter IDUS probe with a frequency of 20-MHz (MAJ-1720, Olympus, Tokyo, Japan) was advanced over a guidewire into the bile duct during the procedure. We used a BLF-600R X-ray machine (Toshiba, Tokyo, Japan) for fluoroscopy. All patients were under general anesthesia in the supine position.

### Operation procedure

The duodenal scope was inserted into the second part of the duodenum. Cannulation of the bile duct was routinely performed. If cannulation time was longer than 15 min, an additional record was made. If the guidewire was inserted into the pancreatic duct, the performer either withdrew the guidewire or applied another guidewire by using the dual guidewire method, which was also reported in written. After successful cannulation, the catheter was advanced to the hilar area; then, cholangiography was done. In the IDUS-ALC group and IDUS-AL group, the amount, shape, and size of stones were defined according to the cholangiography results. In the IDUS-BL group, IDUS was done after cholangiography, and the amount, shape, and size of stones were recorded according to the cholangiography or/and IDUS results. The location of stones was defined according to different regions (hilar, middle, and lower part of CBD). Sphincterotomy was then carried out, and endoscopic papillary balloon dilation (EPBD) was determined according to the size of the stones. The application of lithotripsy, basket, or balloon was depended on the specific case. When the calculus was taken out, the actual size and amount of the stones were recorded. The number of stones was then graded: one and two stones were defined as score 1 and score 2, respectively; >2 stones were described as score 3. A score of sludge was defined according to the times of pulling out with basket or balloon as mentioned before. The size of the stone was measured according to the dimension of the instruments. After exaction of the stone, full clearance was confirmed by cholangiography or/and IDUS in different groups. Nasobiliary drainage was placed in all patients. The fluoroscopy time was also recorded after the operation.

### Outcomes and follow-up

A routine blood test was carried out afterward. Possible complications were recorded, including hemorrhage, perforation, and acute pancreatitis. Nasobiliary cholangiography was performed within 72 h after the operation, and the abdominal US was performed within 1 week to see if there is residual of stones. Patients were followed up on 3, 6, and 12 months after discharge.

### Statistical analysis

Numerical data were expressed as mean ± SD. Continuous variables were compared using Student’s *t*-test and the Mann–Whitney U test, and multiple comparisons were performed using the Kruskal–Wallis test and ANOVA with a Bonferroni correction using SPSS 13.0. *P* < 0.05 was considered to be statistically significant. Categorical variables were evaluated using Pearson χ^2^ test and Fisher exact test.

## Results

### Baseline patients characteristics

No difference in baseline characteristics, including chronic commodities and history of biliary operation, were observed among the three groups (all *P* > 0.05) (Table 1).

**TABLE 1:** Baseline patient characteristics.

Variable	IDUS-BL *n* = 61	IDUS-ALC *n* = 62	IDUS-AL *n* = 61	*P*-value
Age	72.13 ± 14.06	72.39 ± 11.26	73.97 ± 15.35	0.724
Sex				0.303
Male	25 (41.0)	34 (54.8)	30 (49.2)	
Female	36 (59.0)	28 (45.2)	31 (50.8)	
**Commodities**				
Hypertension	21 (34.4)	23 (37.1)	16 (26.2)	0.409
Diabetes	7 (11.5)	10 (16.1)	12 (19.7)	0.460
Cerebral infarction	1 (1.6)	2 (3.2)	3 (4.9)	0.595
Coronary heart disease	7 (11.5)	6 (9.7)	7 (11.5)	0.934
Chronic liver or kidney disease	10 (16.4)	16 (25.8)	10 (16.4)	0.314
Total	32 (52.5)	36 (58.1)	29 (47.5)	0.505
**History of ERCP**				
Yes	18 (29.5)	21 (33.9)	19 (31.1)	0.871
No	43 (70.5)	41 (66.1)	42 (68.9)	
**History of cholecystectomy**				
Yes	15 (24.6)	21 (33.9)	10 (16.4)	0.081
No	46 (75.4)	41 (66.1)	51 (83.6)	

IDUS-BL, IDUS was performed before lithotomy; IDUS-ALC, cholangiography and IDUS were done after lithotomy; IDUS-AL, only IDUS was done after lithotomy; ERCP, data were expressed as *n* (%).

### Baseline stone characteristics, procedural characteristics, anatomy, and outcomes

No significant difference in the location, max size, and scores of amount of calculus were observed among the three groups (all *P* > 0.05). The diameter of CBD and the existence of diverticulum nearby the major papilla were compared, and no significant difference was found among the three groups (all *P* > 0.05).

Complicated management of stones such as lithotripsy was conducted in a balanced way in different groups. No serious complication was observed after lithotomy among the three groups. Mild pancreatitis rarely occurred.

The fluoroscopy time of the IDUS-AL group was significantly shorter than that of the IDUS-BL and IDUS-ALC group (*P* < 0.05). No residual of the calculus was found in any of the groups during the follow-up period (Table 2).

**TABLE 2:** Baseline stone characteristics, procedural characteristics, anatomy, and outcomes.

	IDUS-BL *n* = 61	IDUS-ALC *n* = 62	IDUS-AL *n* = 61	*P*-value
**Stone characteristics**				
**Location of calculus**				
Hilar	8 (13.1)	11 (17.7)	7 (11.5)	
Middle of CBD	36 (59.0)	37 (59.7)	36 (59.0)	0.812
Lower part of CBD	36 (59.0)	41 (66.1)	46 (75.4)	
**Max size of calculus**				
>1 cm	49 (80.3)	51 (82.3)	48 (78.7)	0.883
≤1 cm	12 (19.7)	11 (17.7)	13 (21.3)	
**Scores of amount of calculus**				
1	20 (32.8)	24 (38.7)	22 (36.1)	
2	25 (40.1)	16 (25.8)	20 (32.8)	0.944
3	16 (26.2)	22 (35.5)	19 (31.1)	
**Anatomy**				
Diameter of CBD	1.88 ± 0.49	1.78 ± 0.37	1.81 ± 0.41	0.424
**Diverticular**				
Yes	21 (34.4)	21 (33.9)	18 (29.5)	0.817
No	40 (65.6)	41 (66.1)	43 (70.5)	
**Procedural characteristics**				
**Lithotripsy**				
Yes	9 (14.8)	11 (17.7)	9 (14.8)	0.871
No	52 (85.2)	51 (82.3)	52 (85.2)	
Fluoroscopy time (min)	2.8 ± 1.2	2.5 ± 1.0	1.5 ± 0.6^a^	0.001
**Outcomes**				
**Complication**				
Hemorrhage	0 (0)	0 (0)	0 (0)	
Perforation	0 (0)	0 (0)	0 (0)	>0.05
Pancreatitis	1 (1.6)	1 (1.6)	1 (1.6)	
Residual of stone during follow-up	0 (0)	0 (0)	0 (0)	>0.05

CBD, common bile duct; FT, fluoroscopy time. ^a^Significantly lower than the other two groups. Data were expressed as mean ± standard deviation (SD) or *n* (%).

### Prejudgment accuracy of stones before lithotomy during endoscopic retrograde cholangiopancreatography and the findings of repeated lithotomy

**FIGURE 2:**
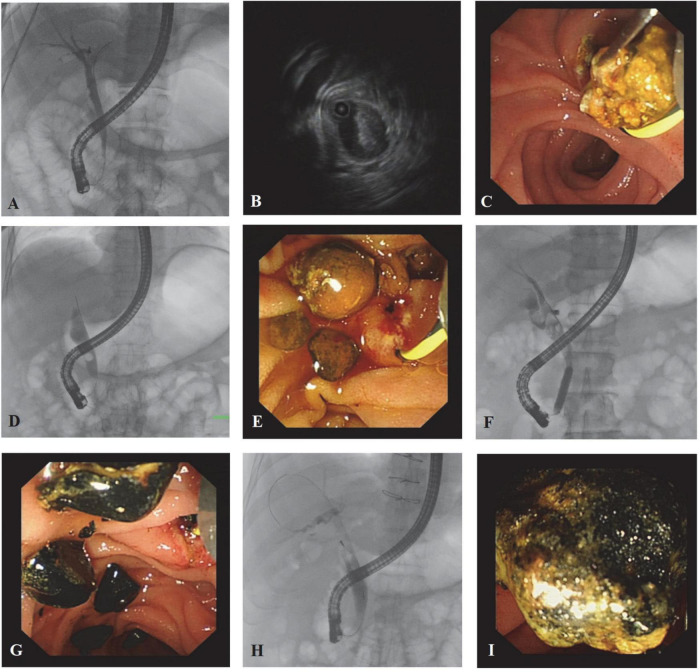
Cholangiographic, IDUS, and endoscopic images from typical cases. Patient 1: A 37-year-old male choledocholithiasis patient was diagnosed with abdominal ultrasonography. **(A)** The cholangiographic image failed to reveal the stones. **(B)** IDUS confirmed the presence of stones in the bile duct, but the number of stones was not accurate. **(C)** During lithotomy, a large amount of sludge was pulled out. Since more than three extractions were done, the score of stones was modified as score 3. Patient 2: A 65-year-old female patient diagnosed with choledocholithiasis by abdominal CT scan. **(D)** Only one stone was found under the shaft of the endoscope, while stones inside the end of the common bile duct could not be seen. **(E)** More than five brownstones were extracted, and most of them existed in the lower part of the common bile duct. Patient 3: A 75-year-old male choledocholithiasis patient was diagnosed with abdominal ultrasonography. **(F)** Cholangiography demonstrated many stones in the middle part of the common bile duct, and stones in the cystic duct can also be seen. **(G)** Black calculus with different sizes was taken out. Patient 4: A 68-year-old male patient was diagnosed with choledocholithiasis by abdominal CT scan. **(H)** Stones <1 cm were shown above the shaft by cholangiography. **(I)** The long diameter of the stone was around 1.2 cm.

**FIGURE 3:**
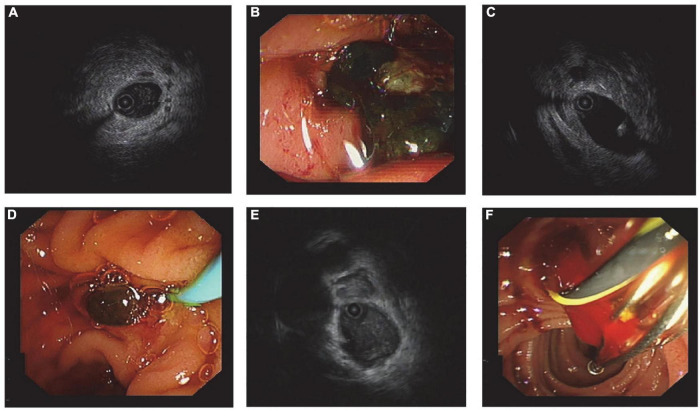
Ultrasonographic and endoscopic images from typical cases. Patient 1: **(A)** IDUS showed flocculent echo in the lumen; **(B)** the thick bile was the cause of acoustic characteristics. Patient 2: **(C)** A little arc high-echo with a shadow was found by IDUS, and it was distinguished from the guidewire, which has ripples in the rear; **(D)** a small stone with a diameter around 3 mm in size was pulled out. Patient 3: **(E)** Flocculent echo was detected inside the bile duct, which resembled the acoustic feature of sludge; **(F)** blood clot was taken out from the common bile duct, and that was because blood stream entered backward into bile duct during sphincterotomy.

Before stones removal, there were a few patients in the three groups whose stones were not accurately evaluated. Both the size and the amount of the stones predicted in advance deviated from the final diagnosis. The accuracy of stone size prediction in the IDUS-BL, IDUS-ALC, and IDUS-AL groups was 86.9% (53/61), 85.5% (53/62), and 88.5% (54/61), respectively, while the accuracy of judging the number of stones in the three groups was 95.1% (58/61), 87.1% (54/62), and 83.6% (51/61), respectively. There was no significant difference among the three groups in the overestimation or underestimation of the size or amount of stones (all *P* > 0.05). Repeated lithotomies were done in 5, 9, and 9 cases of IDUS-BL, IDUS-ALC, and IDUS-AL group, respectively. No statistical difference was found in the frequency of RL and finding of RL (> or <3 mm calculus) among the three groups (Table 3 and Figures 2, 3)

**TABLE 3:** Prejudgment accuracy of stones before lithotomy during ERCP and the findings of repeated lithotomy.

	IDUS-BL *n* = 61	IDUS-ALC *n* = 62	IDUS-AL *n* = 61	*P*-value
Accuracy on size	53 (86.9)	53 (85.5)	54 (88.5)	0.882
Inaccuracy on size	8 (13.1)	9 (14.5)	7 (11.5)	
Overestimated	2	3	2	
Underestimated	6	6	5	
Accuracy on amount	58 (95.1)	54 (87.1)	51 (83.6)	0.124
Inaccuracy on amount	3 (4.9)	8 (12.9)	10 (16.4)	
Overestimated	1 (blood)	0	1 (bubble)	
Underestimated	2	8	9	
Repeated lithotomy	5 (8.2)	9 (14.5)	9 (14.8)	0.462
Stone >3 mm	3	5	6	
Stone <3 mm	2	3	2	
No stone	0	1	1	

IDUS-BL, IDUS was performed before lithotomy; IDUS-ALC, cholangiography and IDUS were done after lithotomy; IDUS-AL, only IDUS was done after lithotomy; RL, repeated lithotomy. Data were expressed as n or *n* (%).

### *Post hoc* analysis: Features of calculus and anatomy in repeated lithotomy patients

A hundred and sixty-one procedures were done in a one-time lithotomy pattern, and 23 cases underwent RL. There was no significant difference in the size and amount of stones in RL cases compared with one-time lithotomy cases (all *P* > 0.05); however, more stones were located in the middle or lower part of CBD in RL cases (*P* < 0.05). In addition, the lithotripsy ratio was higher in patients with RL (*P* < 0.05). The existence of the diverticulum was comparable in one-time lithotomy cases and RL cases. The diameter of RL cases was significantly larger than in one-time lithotomy cases (*P* < 0.05). RL cases had a significantly higher inaccuracy prejudgment ratio before lithotomy than others (*P* < 0.05) (Table 4).

**TABLE 4:** *Post hoc* analysis: features of calculus and anatomy in RL patients.

	One-time lithotomy 161	Repeated lithotomy 23	*P*-value
**Size of calculus**			0.462
>1 cm	125 (77.6)	23 (100)	
<1 cm	36 (22.4)	0 (0)	
**Location of calculus**			0.041
Hilar	26 (16.1)	0 (0)	
Middle of CBD	87 (54.0)	22 (95.7)	
Lower part of CBD	100 (62.1)	23 (100)	
**Scores of the amount of calculus**			
1	62 (38.5)	4 (17.4)	
2	52 (32.3)	9 (39.1)	
3	47 (29.2)	10 (43.5)	0.100
Lithotripsy			<0.0001
Yes	11 (6.8)	18 (78.3)	
No	150 (93.2)	5 (21.7)	
Diverticulum			0.635
Yes	54 (33.5)	6 (26.1)	
No	107 (66.5)	17 (73.9)	
Diameter of CBD	1.78 ± 0.40	2.12 ± 0.46	0.0002
Inaccuracy prejudgment before lithotomy			<0.0001
Yes	6 (3.7)	18 (78.3)	
No	155 (96.3)	5 (21.7)	

RL, repeated lithotomy. Data were expressed as mean ± standard deviation (SD) or *n* (%).

## Discussion

As a high-frequency and highly sensitive examination method, IDUS has important clinical value in diagnosing the biliary tract and pancreatic duct stenosis. Some studies suggested that IDUS is more accurate than EUS, transpapillary biopsy, and cell brushing for detecting biliary tract malignant lesions (12, 13). IDUS is relatively sensitive and specific in differentiating cholangitis from early cholangiocarcinoma (14). Moreover, previous studies have suggested that IDUS increases the diagnostic sensitivity and the specificity for primary sclerosing cholangitis from 62.5 to 87.5% and 53.1 to 90.6%, respectively, and the diagnostic accuracy of malignant lesions from 55 to 90% (15). Because of the bile component in the bile duct as a medium, ultrasound has good conductivity. So far, several studies have reported on IDUS for the diagnosis of CBD stones (16, 17). For example, Kim et al. (18) found that IDUS is useful for detecting occult CBD stone on ERCP in icteric patients with highly suspected CBD stones. The results showed that stones or sludge were found in all cases, with an average diameter of 2.9 mm. Another study suggested that the sensitivity of ERCP for the diagnosis of stones was significantly affected by the size of stones; the diagnostic rate was decreased in patients with stone size below 8 mm. Endo et al. (19) suggested that if the diameter of the bile duct is larger than 12 mm, routine IDUS examination should be performed. However, there are no more reports about the reasonable intervention time of IDUS in stone extraction. On the one hand, the traditional ERCP can be smoothly achieved with the help of cholangiography, and IDUS is not required for the operation process. On the other hand, there is always a certain rate of residual stones in patients who undergo ERCP. In fact, early stone recurrence after ERCP is often associated with stone omission (8).

In this study, we applied IDUS before or after ERCP. IDUS performed before ERCP can provide additional information about the calculus. After lithotomy IDUS was applied as a double check method of complete clearance of stones. Our results showed that the fluoroscopy time of the IDUS-AL group was significantly lower compared to the IDUS-BL and IDUS-ALC groups due to the reduction of one post lithotomy fluoroscopy. However, the degree of complete stone clearance was not affected. IDUS before lithotomy can give a primary impression of the stones and is helpful for lithotomy. Yet, in this study, we found no significant difference in the accuracy rate for the estimation of stone size and number between the three groups. Therefore, whether IDUS should be applied as a routine examination before stone extraction needs to be further investigated. As a point of view, patients with high risk of stone residual should have IDUS confirmation.

The quality of cholangiography can be influenced by the concentration of contrast and the unintended air in CBD. The personal experience of the operator may also affect the quality of the image. The infusion of contrast into hilar is a challenging process, which can affect the detection of stones. Detection of stones behind the endoscopic shaft or at the end of CBD also depends on the operator’s rich experience. Theoretically, IDUS can show the stones behind the shaft and at the end of the CBD more clearly than radiography, but the parapapillary diverticulum can also present as occupying lesion image under IDUS. Although it is difficult to fill the hilar with contrast, the contrast defect does not hinder the judgment of calculus. Our results also showed that in RL cases, the location of the stones was in the middle or lower part of CBD.

Generally speaking, no residual stones were found during follow-up in the three groups, and no serious complications occurred. Compared with other researches, we achieved a lower complication rate, which can be partly attributed to the assistance of general anesthesia and proper indication for ERCP. The shorter average FT can be due to the personal experience of the operator. Moreover, RL during ERCP was not emphasized by other researchers. Our study showed that RL was related to the accuracy of stone prejudgment. Inaccurate prejudgment led to a higher RL rate. Also, both lithotripsy and bile duct diameter were relevant to RL. After lithotripsy, many stone fragments were produced, so it is difficult to judge whether all the stones were removed. Previous studies have suggested that biliary stenting after lithotripsy is safe, which is consistent with our experience (20). When the bile duct is too wide, it can lead to inadequate angiography, making the basket empty out and the balloon not fit the bile duct wall. The high sensitivity of IDUS makes it possible to detect stones 1–2 mm in size. We are also concerned about whether the stones removed in RL are larger than 3 mm, because stones larger than 3 mm might cannot be automatically discharged. The high sensitivity of IDUS may also lead to misjudgment. Thick bile, guidewire, blood clot, and bubbles need to be differentiated from stones (Figure 3).

A few studies suggested that IDUS can reduce the residual rate of stones after ERCP. The residual stones missed by balloon occlusion cholangiography can be washed out with saline (10, 21). This raises another question, i.e., after sphincterotomy, even if stones with a 2–3 mm diameter are missing, can these missing stones flow out with bile. However, another prospective study found that additional IDUS can significantly reduce the stone recurrence rate three years after sphincterotomy (22). The analysis of risk factors for stone recurrence showed that patients with non-calculus gallbladder had low stone recurrence rate due to the flushing effect of bile flow.

This study has a few limitations. (1) There was heterogeneity among total patients according to the exaction methods of stones. Differences in the application of basket and balloon might influence the operation time or FT. After RL, patients did not receive further confirmation of total clearance. (2) No control group (e.g., patients who did not receive IDUS) was selected. IDUS is a time-consuming procedure, although it affects the operation time, it does not rely on fluoroscopy, so it does not affect the fluoroscopy time. IDUS showed a high sensitivity for detecting calculus but was not very effective for quantitative analysis of the number of stones, which can be partially attributed to our score system. The grading system for stones was developed based on the difficulty of lithotomy and the length of operation time. Although it showed to be sufficient for the evaluation of stone clearance, it can cause misjudgment for IDUS.

It was found that no matter IDUS was performed before stone removal for pre-judgment, or IDUS evaluation after stone removal, complete stone removal was completed without complications. After stone removal, IDUS can alone be used to confirm whether the stone is completely removed, reducing the radiation time. In summary, although prejudgment can simplify the process of lithotomy, the time and way of using IDUS need to be considered. Our data suggested that IDUS examination around lithotomy is helpful for the complete and safe extraction of stones. IDUS can also reduce fluoroscopy time and the rate of RL. Since IDUS can detect minimal stone in CBD, it can be potentially used as a golden standard for a total clearance of stones.

## Data availability statement

The raw data supporting the conclusions of this article will be made available by the authors, without undue reservation.

## Ethics statement

The studies involving human participants were reviewed and approved by the Ethics Committee of Shanghai First People’s Hospital. The patients/participants provided their written informed consent to participate in this study.

## Author contributions

HZ was responsible for the design of the subject and the revision of the article. ZJL was responsible for the collection and selection of cases and the writing of the article. Both authors contributed to the article and approved the submitted version.
